# In Vitro Surface Investigation of Calcium Fluoride-like Precipitation on Human Enamel after Topical Treatment with the Organic Fluoride Nicomethanol Hydrofluoride

**DOI:** 10.3290/j.ohpd.b898957

**Published:** 2021-01-26

**Authors:** Andreas Kiesow, Nico Teuscher, Maria Morawietz, Sigrun Eick

**Affiliations:** a Research Group Leader, Fraunhofer Institute for Microstructure of Materials and Systems IMWS, Halle, Germany. Conceived and designed the experiments, wrote the manuscript, read and approved the final version of the manuscript.; b Research Engineer, Fraunhofer Institute for Microstructure of Materials and Systems IMWS, Halle, Germany. Performed electron microscopy and EDX analysis on the cross sections, contributed to writing the manuscript, read and approved the final version of the manuscript.; c Research Engineer, Fraunhofer Institute for Microstructure of Materials and Systems IMWS, Halle, Germany. Conceived and designed the experiments, wrote the manuscript, read and approved the final version of the manuscript.; d Professor, Department of Periodontology, University of Bern, Bern, Switzerland. Contributed to the planning of the study, assessment of results, and writing of manuscript, read and approved the final version of the manuscript.

**Keywords:** amine fluorides, calcium fluoride precipitation, enamel, energy dispersive x-ray analysis, scanning electron microscopy

## Abstract

**Purpose:**

The topical fluoride treatment of teeth can lead to a formation of CaF_2_-like material, which is considered to play a significant role in caries prevention. Different types of fluoride sources are applied. The aim of this study was to analyse the in vitro fluoridation effect of the lesser known organic fluoride compound nicomethanol hydrofluoride (NH) regarding fluoride accumulation and morphological changes on dental enamel surfaces.

**Materials and Methods:**

The fluoridation effect was investigated by scanning electron microscopy (SEM) and energy dispersive x-ray analysis (EDX) after treatment with fluoride solutions at a concentration of 1350 ppm F ^-^ and a pH value of 5.5. NH was tested against inorganic sodium fluoride (NaF) as reference. Fluoridation was done on pellicle-free and pellicle-covered enamel.

**Results:**

Formation of globular CaF_2_-like material was observed for both fluoride types. However, NH led to considerably higher calcium fluoride accumulation on the enamel surface as shown by both EDX and SEM. The globule diameters varied between 0.2 and 0.8 µm. Cross-sectional analysis revealed that the globular precipitates lay directly on the enamel surface; only the very surface-near volume was affected. No statistically significant difference of the fluoridation effect was measured with vs without saliva pre-treatment.

**Conclusion:**

The experiments showed a 6 times greater F ^-^ surface uptake on dental enamel with NH compared to sodium fluoride, thus suggesting an important role of NH during remineralization phases, fostering equilibrium between de- and remineralization.

The topical application of fluoridated oral care (OC) products is well-established for caries^1,18,21,25,27,28^ and erosion^2,6,10,11,27^ protection. Regarding the mode of action, it is well known that under certain conditions, a calcium fluoride (CaF_2_)-like material can be formed on the tooth surface,^8^ and this may be one of the main explanations for the protective effect seen following the treatment of enamel with acidic fluorides.^5^ The formed calcium fluoride is an important source of fluoride for the oral fluids and it is known as a pH-controlled reservoir of fluoride and calcium.^1,18^ Beside caries and erosion protection, the beneficial role of fluorides in the preventive management of dentinal hypersensitivity is described in the literature.^15^

A variety of fluoride compounds is used in OC products. Due to differences in their molecular structure, not all fluoride compounds are similarly efficacious in interacting with the enamel or dentin surfaces (i.e. they can have different influences on the mode of action^14^). The three dominant inorganic fluorides used in toothpastes worldwide are: sodium fluoride (NaF) and stannous difluoride (SnF_2_), both are readily soluble metal salts, and sodium monofluorophosphate (Na_2_FPO_3_), in which fluoride is covalently bound to the phosphate group and hydrolysis is required to release fluoride ions.^9,14^

In addition to the inorganic fluorides, organic fluorides are a further fluoride source. Here, the amine fluorides (AmF) are the most well-known and dominating group.^13^ Amine fluorides are characterized by specific surface properties compared to inorganic fluorides. They possess organic cations with a bipolar molecular structure which acts as a counter ion to fluoride. Due to their amphiphilic molecular structure, they have relatively high surface activity and attach to the enamel surface via the polar amine moiety. It is this effect that allows AmF to function as ”fluoride carriers” to the enamel surface and consequently induce the formation of the CaF_2_-like material.^24^

Olaflur and dectaflur belong to the group of amine fluorides and are possibly the best-known representatives of this group in dentistry. Compared to these amine fluorides, relatively little has been published about the organic amine fluoride nicomethanol hydrofluoride (NH; Pierre Fabre patent 75.23715). NH is distributed under the product name Fluorinol. As an ingredient in different OC products it has been launched in several countries over the last decade.^7^ Although studies^7,24,26^ are available in which NH has been investigated regarding its protective effects against dental caries, knowledge is still needed to understand the reaction processes of NH on dental hard tissue. In particular, it is of interest to understand more about the interaction of NH with the enamel surface in regard to morphological and chemical changes after application. Microstructure and composition of calcium fluoride precipitate have been investigated intensively for enamel treatments with NaF and Olaflur solutions.^5,12,16,17,20^ According to the best of the authors’ knowledge, there are no published studies characterizing the formation of CaF_2_-like material after treatment with NH using microscopic techniques.

The aim of this in vitro study was to analyze the fluoridation effects of the organic fluoride compound NH on dental enamel by scanning electron microscopy (SEM) in combination with energy disperse x-ray (EDX) analysis. The focus of this investigation was on the microstructural description of the fluoridation effects, i.e. the characteristics of the reaction products deposited on the surface and the way the surface’s near volume is affected, as well as on obtaining quantitative data about the chemical changes on the surface.

## Materials and Methods

### Preparation of the Enamel Samples

From a pool of extracted teeth, a total of ten caries-free mandibular molars were selected. Before the extraction, the patients were informed about the use of their teeth for research purposes and their spoken consent was obtained. This procedure complies with the approved guidelines and regulations of the local ethics committee (Kantonale Ethikkommission KEK) for irreversibly anonymized samples. The teeth were stored immediately after extraction in an ethanol (5%)/thymol (0.5%) solution. The teeth were sectioned using a water-cooled, diamond-bladed saw (Presi Minitech 333, PRESI; Grenoble, France) into similar-sized specimens. The enamel specimens were embedded in epoxy resin (SpexiFix20, Struers; Ballerup, Denmark) and successively wet ground with silica carbide paper (120-4000 grit, Struersresulting an even sample surface. Finally, the surface was slightly etched by 1% citric acid (pH 3.8) for 15 min to remove the upper surface layer affected by polishing, and the teeth were stored in 0.9% w/v NaCl solution for about two weeks. Then, the samples were randomly divided into four treatment groups. Five specimens per treatment group were investigated.

For creating a pellicle layer, the enamel samples were stored in pooled human saliva (non-stimulated, pooled from 3 people) for 2 h at 37°C and subsequently rinsed with deionized water. Saliva sampling was also carried out in accordance with the approved guidelines and regulations of the local ethics committee (KEK). The volunteers were informed about the use of their saliva in research, and their spoken consent was obtained. Because the saliva was pooled, the ethics committee classified it as “irreversibly anonymized”, and therefore, no previous ethical approval from the KEK was necessary.

### Fluoride Solutions

Two different test fluoride solutions were included in the study. Nicomethanol hydrofluoride (F^-^ concentration of 20%) was provided as aqueous stock solutions by Pierre Fabre Laboratories (Castres, France). Sodium fluoride was purchased from Sigma Aldrich (Sigma-Aldrich; St Louis, MO, USA). The NH solution was prepared by diluting the stock solution of NH with acetic acid at pH 5.5 to a F^-^ concentration of 1350 ppm. NaF test solution was prepared by dissolving NaF powder in acetic acid at pH 5.5, thus also obtaining a F^-^ concentration of 1350 ppm. The fluoride concentration was controlled by a fluoride-sensitive electrode (DC219-F, Mettler-Toledo; Columbus, OH, USA). The pH value of 5.5, measured by a pH meter (Multi seven, Mettler-Toledo) at room temperature, was controlled and adjusted in final fluoride solutions. pH values of the resulting solutions were adjusted (if necessary) to 5.5 by addition of either sodium hydroxide (NaOH) or nitric acid (HNO_3_). The fluoridation experiments were performed at a stable pH value.

### Fluoride Application

The study included four treatment groups:

- Group NaF-n. 2 min NaF application on native enamel samples
- Group NH-n. 2 min NH application on native enamel samples
- Group NaF-s. 2 min NaF application on enamel samples pre-treated with saliva
- Group NH-s. 2 min NH application on enamel samples pre-treated with saliva

Enamel specimens were exposed for 2 min to the respective test solutions by submerging them in a beaker containing 20 ml of the test solutions at room temperature under constant, gentle agitation at 100 rpm (KS 260 Basic; IKA Werke: Staufen, Germany). Care was taken that the relevant surface of the specimen was exposed to the ambient fluoride solution. After exposure, the specimens were removed from the test solution and rinsed under running deionized water for about 3 s. After the rinsing step the samples were prepared for microscopic analysis.

### Scanning Electron Microscopy and Energy Dispersive X-ray Analysis

The surface of all enamel specimens was examined by SEM to characterize surface morphology and by EDX to quantify chemical surface changes, in particular to determine the fluoride content, due to the fluoride exposure. SEM and EDX analyses were performed using a JSM 7401F, (JEOL; Tokyo, Japan) equipped with an EDX detector (Genesis; EDAX; Mahwah, NJ, USA). Prior to the analysis, the samples were surface coated with palladium (Pd) to counteract electrical surface charging during analyses. SEM measurements were performed on two positions at two different magnifications (10,000X, 30,000X). EDX measurements were performed on one representative position per sample. EDX measurements were made by integral-analysis over the recording window at a magnification of 10,000X. Statistical analysis of data from the EDX analysis were made by one-way ANOVA with post-hoc Tukey’s test. Differences with a p-value < 0.05 were considered statistically significant.

### Analysis of Cross Sections

SEM and EDX investigations on cross sections were performed only on samples for treatment group NH-s. A SUPRA 55VP microscope (Zeiss; Jena, Germany) with Octane Elite 30 mm^2^ EDX system plus TEAM-Software (EDAX; Mahwah, NJ, USA) was used for EDX line profile analysis on single globular precipitates. To obtain longitudinal sections of the fluoride-treated samples, the enamel specimens were cut into halves, followed by embedding one half in epoxy resin (SpexiFix20, Struers). The resulting cross-sectional surface was prepared by grinding as described above.

## Results

### EDX Analysis

**Fig 1:**
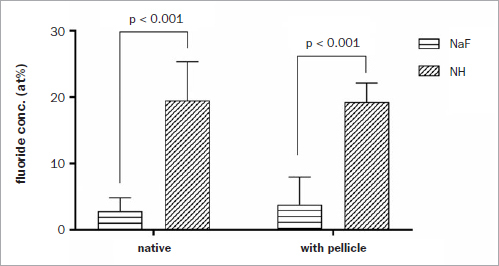
Mean fluoride content for the four treatment groups determined by surface EDX measurement. There was no statistically significant difference between treatment groups NaF-n and NaF-s, or between treatment groups NH-n and NH-s.

The fluoride content of the treated samples as measured by the EDX surface analysis is presented in Fig 1. Pre-coating the enamel specimens with saliva obviously did not affect the fluoride uptake, as there was no statistically significant difference between the fluoride concentrations for treatment groups NaF-n and NaF-s or between treatment groups NH-n and NH-s. The fluoride content was statistically significantly higher (5-fold to 7-fold) for the samples treated with NH (groups NH-n, NH-s) in comparison to the samples treated with NaF (group NaF-n, NaF-s).

A fluoride content of 0.08 at% (atomic percent) was measured for an untreated enamel sample, which is below the detection limit of the method.

### SEM Results

**Fig 2:**
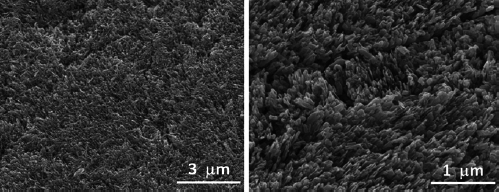
SEM micrographs demonstrating an enamel surface prior to fluoridation. The surface is characterized by needle-like structures of hydroxyapatite crystallites exposed due to acid pre-treatment.

**Fig 3:**
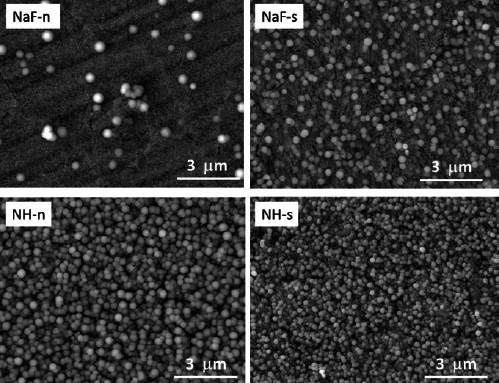
Representative SEM micrographs (10,000X magnification) demonstrating calcium fluoride precipitation according the different treatment groups. The micrographs give an overview of the distribution and density of the globular precipitates over the enamel surface.

**Fig 4:**
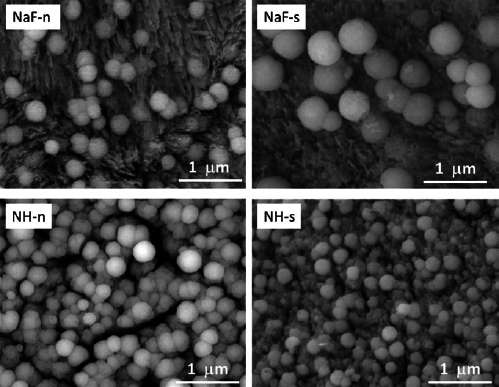
Representative SEM micrographs (30,000X magnification) demonstrating calcium fluoride preciptiation in the different treatment groups, showing the globular shape, size of the precipitates and how they lie on the surface.

SEM investigations revealed that globular precipitated structures were generally found on the surface for all specimens treated with the fluoride solution, both for the NaF and for the NH treatment. An untreated sample was completely free of such globular precipitates, as seen in Fig 2. SEM micrographs at lower magnification (Fig 3) give an overview of the distribution of the globular precipitates across the enamel surface for all four treatments. The treatment with NH solution (treatment groups NH-n, NH-s) leads to a distinctly higher precipitation rate compared to the treatment with NaF solution (treatment groups NaF-n, NaF-s), i.e. the surface is characterized by a dense layer of globular precipitates which covers the surface almost completely. These globular structures can be assumed to be CaF_2_-like precipitates. In contrast, the CaF_2_-like globules are scattered on the surface and available in much smaller quantities for the NaF treatments. Due to the lower density of the precipitates, the needle-like hydroxyapatite structures are obvious in Fig 4 (NaF-n, NaF-s). Generally, the precipitates of different treatments do not show statistically significant differences regarding shape or other morphological properties. The diameter of the CaF_2_-like globules is in the range of 0.2 to 0.8 µm. Correlations between globule diameter and treatment type are not obvious. The morphology of the globular structures is of the same nature for the NH and NaF treatments. Also, no trend could be observed comparing specimens which were pre-treated with saliva or not pre-treated.

### Cross Sections of Enamel Samples Precoated with Saliva and Treated with NH

**Fig 5:**
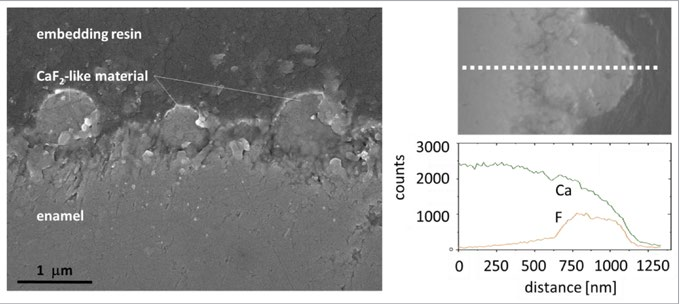
Left: Cross-sectional SEM image of an enamel specimen coated with saliva and treated with NH. CaF_2_-like globules are located apart from each other on the enamel surface. This contrasts slightly with the micrographs in Figs 3 and 4, NH-n and NH-s), suggesting a much higher packing density for NH treatments. Right: SEM image of one calcium fluoride globule and respective EDX line profiles for F and Ca (signal intensity in counts) of the same sample. There is some fluoride below the globule, which however decreases with increasing depth and reaches the EDX detection limit at a depth of around 600 nm.

In Fig 5 (left), a cross section of a sample treated with NH is shown. The almost spherical shape of the precipitates is illustrated by three CaF_2_-like globules. These precipitated reaction products sit directly on the enamel surface. Their spherical shape is slightly disturbed at the interface between enamel and globular structure. Some preparation artefacts in the form of debris are visible on the margin of the globules. The interface is characterized by a small fuzzy transition region connecting both materials: CaF_2_-like material and human enamel. The surface region between the globules is not even; it is slightly disturbed by the demineralization step prior to the treatment, and possibly also by the acidic fluoride treatment.

Figure 5 (right) shows EDX line (depth) profiles for the elements F und Ca measured on a cross-sectioned specimen starting in the sound, unaffected enamel and going to the top of the globular precipitate. The F-intensity profile corresponds very well with the morphology of the globular CaF_2_-like material. Following the F^-^ profile into the underlying enamel, the fluoride concentration decreases quickly. The limit for F-detection is reached only after a few hundred nanometres from the surface (depth of about 600 nm).

## Discussion

The present study was designed to examine nicomethanol hydrofluoride prepared in a moderately acidic fluoride solution (pH 5.5) regarding CaF_2_-like formation on dental enamel and to test its activity in comparison with a comparable sodium fluoride solution. Dental enamel was exposed to pure fluoride stock solutions in the experiments in order to limit the complexity of the reactions between enamel and fluoride and to be comparable with existing studies. Regarding the design, the present study followed Sharkov’s basic approach^24^ in comparing treatment results of NH with NaF (as benchmark), except for the detection of calcium fluoride for which they used IR-spectroscopy. In the present study, SEM and EDX were chosen for this investigation, since these methods are well established for characterizing fluoride-enamel interactions and have been used in similar studies characterizing other fluoride compounds, e.g. titanium tetrafluoride, stannous fluoride, and AmF.^5,16,20,23^

Based on the obtained results, it can be concluded that the globular reaction products on the enamel surface observed after treatment with NH solution are CaF_2_-like material. These results and the comparison with literature data suggest that the fluoridation effects of NH are of the same kind as observed for NaF or the amine fluoride Olaflur.^16^ To the authors’ best knowledge, the morphological characterization of the CaF_2_-like precipitates generated by NH treatment has not been not reported elsewhere in the literature.

The morphological appearance and size of the CaF_2_-like globular precipitates is not statistically significantly different for the NaF and NH treatments. The size of globules varied between a few hundred up to 600 nm maximum. This order of magnitude corresponds well with results by Petzold^16^ (NaF, AmF [Olaflur]) and Rošin-Grget^20^ (AmF; N, N, N’-3-bihydroxyethyl-N’-octadecyl-1,3-diaminopropan-dihydrofluoride). Since the globular precipitates show identical morphological properties, it can be assumed that a similar or even identical fundamental reaction mechanism, based on free fluoride ions from the fluoride solution and dissolved minerals (calcium) from the enamel, is responsible for the formation of the individual globular precipitates. This is supported by a study by Petzold^16^ in which no statistically significant differences were found for the chemical composition of calcium fluoride globules generated by AmF (Olaflur) or NaF treatments. Transmission electron microscopy observations found a polycrystalline microstructure having grain sizes ranging between about 2 and 20 nm for the globular calcium fluoride precipitates.^17^

In the present study, fully packed, layer-like surface coating with globular calcium fluoride precipitates was observed only for samples exposed to NH, not for NaF. This is also quantitatively reflected by the EDX data. The obviously much faster surface reaction by NH resulted in an approximately six times greater fluoride content on the surface. This finding is in agreement with reports by Sharkov^24^ and Lacout,^7^ which however, used other analytical approaches to determine fluoride uptake.

Generally, increasing deposition of calcium fluoride can be achieved by extended exposure times, increased fluoride concentration or by lowering the pH.^19,20^ Since all of these parameters were the same for the NH and NaF treatments, the differing content of calcium fluoride formation can only be explained by different reaction modalities in the presence of AmF molecules compared to NaF. Due to the specific molecular structure of NH with a hydrophilic part (amine group) and a hydrophobic part (hydrocarbon chain), the surface tension of the solution increases, which is associated with increased affinity of F-ions for the enamel surface.^24^ The F ^-^ ions might be available more quickly to be exchanged with OH-groups from the hydroxyapatite of enamel or even more quickly for the reaction with the dissolved free Ca^2+^ to form CaF_2_-like material.^24^ This study may have been limited by the fact that only NaF was included as a comparison, and no other AmF was included.

Further, the samples were only analyzed immediately after exposing them to fluoride solutions for 2 min. Determining calcium fluoride after eluting samples in saliva for several hours might be also of interest. Analyzing the fluoride content on enamel surfaces after application of an amine fluoride (Olaflur) gel and a NaF gel confirmed a higher uptake after 1 h, whereas at later timepoints, the amount decreased to a level at which the two fluorides no longer differed.^4^

In order to learn more about the vertical surface-near structure, one NH-treated sample was prepared accordingly and investigated in cross section (Fig 4). From the morphological point of view, it can be assumed that the reaction occurs directly on the enamel surface and deeper regions remain unaffected. The relatively small depth of interaction is supported by the EDX-line profile for F and Ca. The line profile progression indicates that the globule structure consists of CaF_2_-like material. The results presented here confirm the findings by Müller et al^12^ regarding the depth of the fluoridation effects, i.e. that the material interactions take place on a nanometer scale. However, regarding the structure of the CaF_2_ layer, we do not suggest a gradual progression consisting of three layers (CaF_2_, Ca(OH)_2_ and fluorapatite [Fap]) as postulated by Müller et al^12^ and Gerth et al.^3^ The findings of the present study indicate a relatively clear interface between the calcium fluoride-like precipitate and the underlying enamel.

In order to examine whether the surface reactivity of fluoride on dental enamel is influenced by saliva, one half of the enamel samples were pre-treated with fresh human saliva to obtain a dental pellicle layer. It was of particular interest to see if the amphiphilic molecular structure of NH shows a specific attraction to the saliva protein layer resulting in a distinct change in surface fluoridation rate. However, the chosen experimental approach was not able to show an influence of the pellicle, be it because such an influence is too small to be detected or even absent. Rošin-Grget et al^22^ reported that saliva pre-treatment could enhance the amount of calcium fluoride; but the authors mentioned further that there is a certain disparity in the scientific literature regarding the findings of in vitro studies. In recent studies,^5,16,20,23^ higher fluoride uptake was measured after treatment with different acidic fluoride formulations on enamel surfaces without saliva pre-treatment. For a better understanding of the pellicle and saliva influence, more complex studies have to be performed. Experimental test set-ups could be expanded by mimicking real oral conditions, i.e. taking into account the fluoride reactions in the presence of saliva.

Regarding the presence of saliva and the applied treatment parameters, this study was designed to obtain basic data about the precipitation behavior of CaF_2_-like material after applying NH and to be comparable to other studies.^5,16,22^ In terms of the application of oral care products and the situation in the oral cavity, it has to be considered that fluoride concentration and pH value change with time due to the existence of saliva. This complex situation was not considered in this study.

In terms of clinical relevance, the results for NH could suggest reducing the fluoride content in oral care products. For that however, more studies are necessary to investigate the bioavailability of fluoride and the clinical efficacy in oral care products. Another interesting point for further studies would be to compare different amine fluorides.

## Conclusion

The application of NH leads to precipitation of CaF_2_-like material on dental enamel. Under the experimental conditions, NH had statistically significantly higher activity regarding CaF_2_-like surface precipitation compared to NaF. In conclusion, since a calcium fluoride-like layer acts as pH-controlled fluoride and calcium-ion reservoir, NH applied topically could play an important and positive role in the dynamic process of de- and remineralization by favorably shifting the balance towards the prevention of demineralization. Studies with more clinically realistic models, such as cyclic de- and remineralization tests or in situ fluoride uptake are needed to fully understand the mechanism of action of NH; in particular, NH applied in oral care products should be investigated further.
